# Far Red‐Shifted CdTe Quantum Dots for Multicolour Stimulated Emission Depletion Nanoscopy

**DOI:** 10.1002/cphc.202200698

**Published:** 2022-11-09

**Authors:** Jonatan Alvelid, Andrea Bucci, Ilaria Testa

**Affiliations:** ^1^ Department of Applied Physics and SciLifeLab KTH Royal Institute of Technology 114 28 Stockholm Sweden

**Keywords:** quantum dots, biophysics, microscopy, multicolour, stimulated emission depletion

## Abstract

Stimulated emission depletion (STED) nanoscopy is a widely used nanoscopy technique. Two‐colour STED imaging in fixed and living cells is standardised today utilising both fluorescent dyes and fluorescent proteins. Solutions to image additional colours have been demonstrated using spectral unmixing, photobleaching steps, or long‐Stokes‐shift dyes. However, these approaches often compromise speed, spatial resolution, and image quality, and increase complexity. Here, we present multicolour STED nanoscopy with far red‐shifted semiconductor CdTe quantum dots (QDs). STED imaging of the QDs is optimized to minimize blinking effects and maximize the number of detected photons. The far‐red and compact emission spectra of the investigated QDs free spectral space for the simultaneous use of fluorescent dyes, enabling straightforward three‐colour STED imaging with a single depletion beam. We use our method to study the internalization of QDs in cells, opening up the way for future super‐resolution studies of particle uptake and internalization.

## Graphical abstract

**Far red‐shifted quantum dots** are used as fluorophores in stimulated emission depletion (STED) imaging. The imaging parameters are optimized to minimize blinking effects and maximize brightness. Their emission spectra allow combination with fluorescent dyes to perform three‐colour STED imaging with a single depletion beam. Using this method, the nanoparticle uptake through the endocytic pathway is investigated.

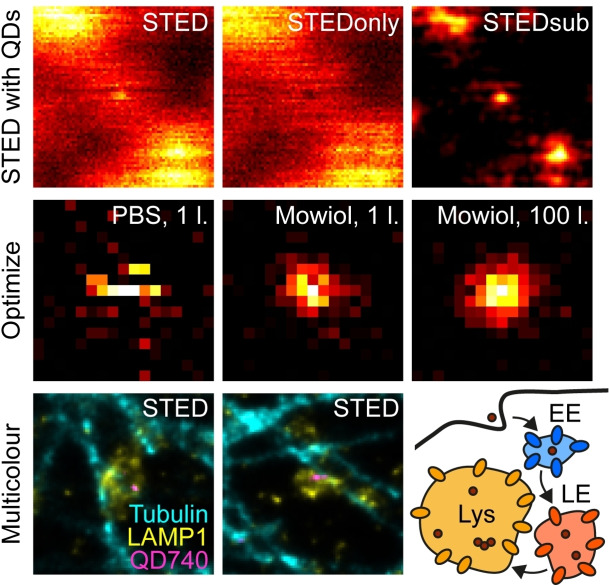

## Introduction

STED nanoscopy[^1^, ^2^] has since its invention been developed in many directions. Together, all the developments have made it one of the most versatile and used nanoscopy methods today for investigation of biological questions,[^3^, ^4^, ^5^] owing to its flexibility, intrinsic optical sectioning, and multicolour capability. STED nanoscopy belongs to the family of coordinate‐targeted nanoscopy techniques which overcome the diffraction limit by confining the fluorescence emission into a small volume. STED does so by using stimulated emission, a process almost universal to fluorophores, initiated by a red‐shifted beam of light. Typically, the depletion beam is a doughnut‐shaped intensity focus, which is formed by imparting a helical phase shift.

Multicolour capabilities of STED nanoscopy most commonly relies on spectral separation of multiple dye species in terms of excitation and emission spectra. In this way reaching two quasi‐simultaneous colours by line‐alternating imaging has been demonstrated both in fixed and live samples.^[6]^ The bottleneck of reaching additional colours with a single depletion beam is related to an intrinsic property of the STED principle: the depletion wavelength must overlap the emission spectra of the fluorophores used to efficiently cause stimulated emission. A wavelength of 775 nm will overlap fluorophores emitting around 670 nm, such as STAR635P, as well as fluorophores emitting around 610 nm, such as ATTO594. However, the use of fluorophores that are further blue‐shifted will inevitably cause a smaller and smaller overlap with the STED wavelength, a loss of depletion efficiency, and hence a loss of resolution. Adding fluorophores with emission spectra between 610 and 670 nm instead causes spectral crosstalk. To avoid these issues, attempts at increasing the number of channels in the final image has been focused on techniques such as spectral unmixing^[7]^ and photobleaching,^[8]^ both reaching up to four channels in the final image. However, spectral unmixing and photobleaching rely on additional processes, complicating the imaging and thus limiting the accessibility to general users. Moreover, post‐processing techniques such as spectral unmixing is potentially error‐prone and needs to be well calibrated. Biological applications will therefore benefit from multicolour techniques based on pure spectral separation in excitation and emission since they are relatively easy to use and can be directly used in existing custom‐built and commercial setups.

A solution to adding additional spectral channels is long‐Stokes‐shift dyes,[^9^, ^10^] where variants exist that emit fluorescence in the far‐red but are excitable at much blue‐shifted wavelengths. Hence, separation can be achieved with the use of distinct excitation wavelengths, but even in this approach linear unmixing is occasionally required.^[9]^ Moreover, the highest resolution shown with long‐Stokes‐shift dyes in a single‐colour STED image is so far 80 nm and in multicolour images even lower,^[10]^ due to the poor photostability of the long‐Stokes‐shift fluorophores currently available.^[11]^ This is significantly lower compared to the performance of organic dyes routinely used in STED microscopy, which can provide a spatial resolution towards 30–40 nm in biological samples. Many long‐Stokes‐shift dyes are also sensitive to the environment, such as the surrounding medium^[12]^ or local pH values,^[13]^ which can cause uneven performance.

On the far‐red end of the spectrum, there is a spectral gap between the emission spectra of STAR635P and the STED wavelength, where a third emission channel could be placed. So far, no successful attempt at filling this gap has been presented, due to the lack of fluorophores both emitting at this wavelength and having a high depletion efficiency. Limitations can also rise from background elicited by direct depletion‐beam excitation of the probe.

The use of nanoparticles as fluorophores for nanoscopy methods have been proposed many times in recent years,^[14]^ but has not reached widespread use for STED so far. This includes quantum dots (QDs),^[15]^ upconversion nanoparticles,[^16^, ^17^] AIE dots,^[18]^ and nanodiamonds.[^19^, ^20^] Most of these probes are not straightforward to use on standard STED microscopes with a depletion wavelength of 775 nm, for reasons including far blue‐shifted excitation and emission,[^18^, ^19^] non‐standardized STED wavelengths,[^16^, ^17^, ^18^] or slow imaging speeds.[^16^, ^19^, ^20^] Moreover, the resolution achieved with many of these alternatives is not close to that achieved with organic dyes, especially in a cellular environment. Lastly, functionalization of nanoparticles is not a standardized process due to the many different materials and surface cappings used, and often requires expert knowledge to carry out. Attempts to standardize the functionalization use extra adaptor molecules such as streptavidin‐biotin attached to antibodies, at the cost of increased linker lengths.^[14]^ Furthermore, few but increasing number of commercially available functionalized nanoparticles do exist, but at a level far from that of organic dyes, increasing the threshold of their usage in nanoscopy.

QDs can be extremely photostable and feature narrow emission spectra, which are appealing properties for multicolour and time‐lapse STED imaging. Previous work has used a 775 nm depletion wavelength with QDs emitting at ∼700 nm,^[15]^ but emission at this wavelength will not be entirely spectrally separable from normally used far‐red emitting organic dyes such as STAR635P, peaking at ∼666 nm. This hinders direct addition of spectral channels to the imaging, but they can be used as a replacement of the common far red‐shifted organic dyes or together with spectral unmixing strategies.

Here, we present the use of QDs emitting at 740 nm for STED nanoscopy with an imaging resolution below 60 nm. This also marks the first time of efficient STED imaging with the 775 nm depletion wavelength of a fluorophore with an emission maximum at a longer wavelength than 720 nm. We also present a straightforward strategy for spectrally separated three‐colour STED imaging with a single depletion wavelength and without additional separation mechanisms for the channels, with potential for sub‐70 nm resolution in all three channels. The approach is directly applicable to commercial as well as custom‐built setups, lending it accessible to widespread use among biological applications.

## Results and Discussion

**Figure 1:**
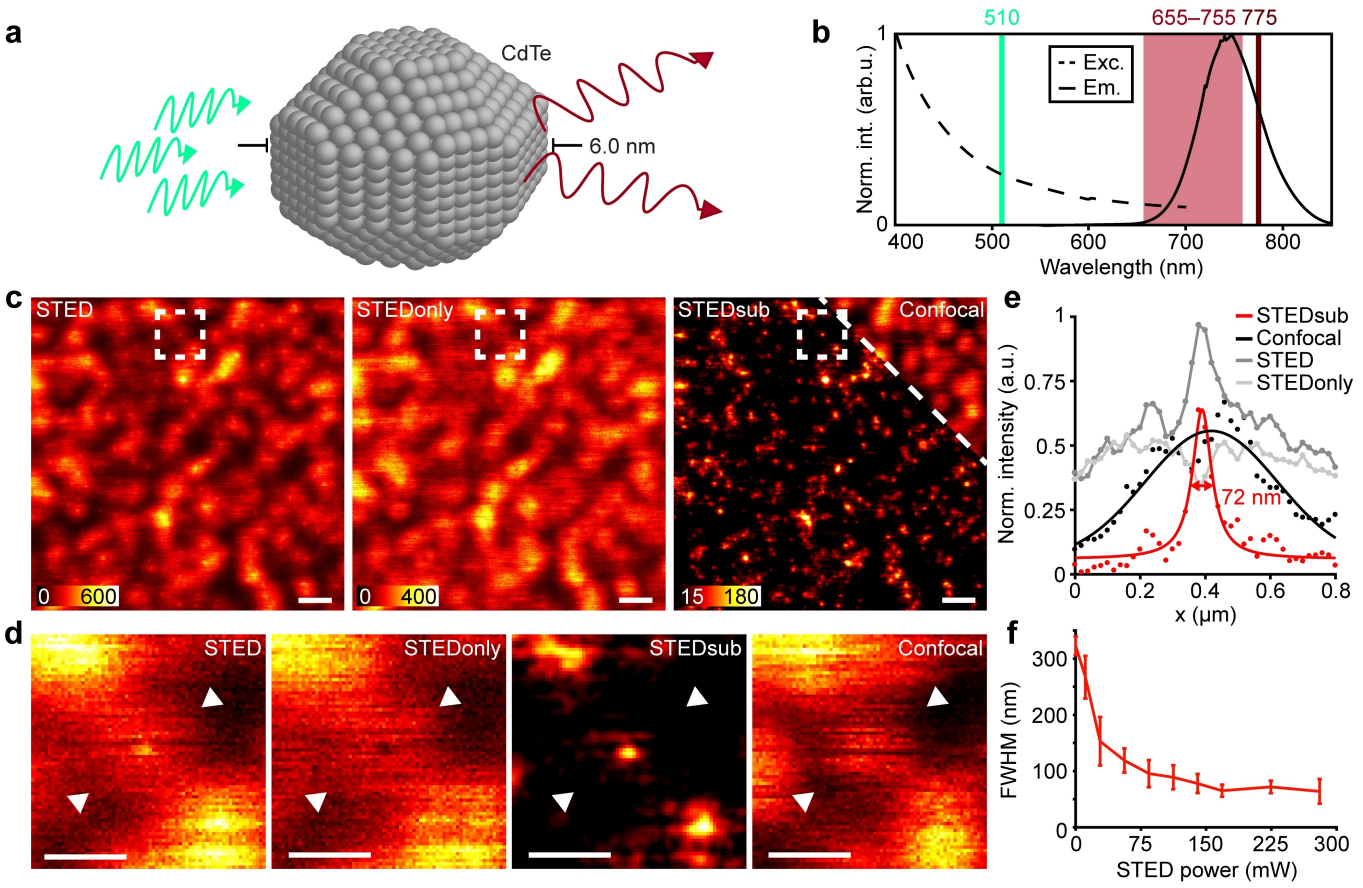
STED imaging of red‐shifted CdTe QDs. (a) Sketch of a 740 nm CdTe quantum dot. (b) Excitation and emission spectra for the 740 nm quantum dots. (c) Resolution improvement of STED performed on QDs, as compared with confocal imaging (I_STED_=0 mW). Recorded images shown are STED, STEDonly, and confocal. STEDsub has been calculated as STED‐STEDonly. Scale bars, 1 μm. (d) Zoom‐ins of a single QD in the region of interest marked in (c), for all the images. Scale bars, 500 nm. (e) Line profile across single QD in (d) (dots) for all the images, and for STEDsub and confocal a fitted Lorentzian (red solid line) and Gaussian (black solid line) respectively. (f) Resolution improvement curve showing the FWHM of QDs in STED images with increasing depletion power. Each point is the average of 10 measured QDs. Error bars indicate 1 standard deviation.

STED imaging is performed on a custom‐built STED microscope^[21]^ based on a 775 nm pulsed picosecond depletion laser. Excitation of the quantum dots and dyes is carried out with three pulsed picosecond lasers at 510 nm, 561 nm, and 640 nm. An extended description of the microscope including hardware and software is presented in the Supporting Information (Supplementary Note S1, S2, Supplementary Figure S3). The QDs used are hydrophilic core‐only CdTe quantum dots with a core diameter of 6.0 nm (Figure 1a) and an emission maximum at 744 nm (Figure 1b).

STED imaging was initially tested on samples with only QDs at the cover glass surface (Figure 1). Exposing the QDs to excitation and depletion beams together in a standardized STED imaging manner, i. e. a train of pulses consisting of a ∼130 ps excitation pulse at 510 nm followed by a delayed ∼530 ps depletion pulse at 775 nm, with detection at 655–755 nm (Figure 1b) generates an image containing the super‐resolution information, but where the QDs have a dimmer fluorescent halo around the super‐resolved centre (Figure 1c, STED). This halo, or background, is caused by direct excitation by the 775 nm depletion beam.^[15]^ After illuminating the sample with the excitation beam, the time‐delayed depletion beam induces stimulated emission after relaxation to the lowest excited vibrational state. However, as the depletion beam continues to illuminate the sample for the pulse width of ∼530 ps, additional photons can interact with the QDs and be absorbed. This will cause direct excitation from higher ground states into the excited state band of energy levels, due to the broad excitation spectrum of the QDs. From here, the excited QD can emit a fluorescence photon of the same energy as if it would have been excited by the 510 nm excitation beam. This secondary fluorescence emission will create a doughnut‐shaped emission in the image, reflecting the shape of the depletion beam. As previous work has introduced,[^15^, ^22^] the full super‐resolved information can be recovered by recording a second image using only the depletion beam, creating doughnut‐shaped emission patterns (Figure 1c, STEDonly), and subtracting this from the original image (Figure 1c, STEDsub). This leads to successful imaging of single QDs (Figure 1d): previously imaged as super‐resolved centres with a doughnut‐shaped halo around it (Figure 1d, STED), they are now imaged as sub‐diffraction‐sized QDs with sizes of ∼70 nm (Figure 1d, STEDsub) and are different compared with confocal images (Figure 1d, Confocal, Figure 1e). The spatial resolution reaches 65±10 nm when using 168 mW depletion illumination power (Figure 1f). This level of depletion power is comparable to that used for organic dyes (∼140 mW),^[6]^ and as such the depletion beam here does not cause any additional phototoxicity to the sample as compared with other STED imaging, rendering it compatible with live‐cell imaging.^[23]^

**Figure 2:**
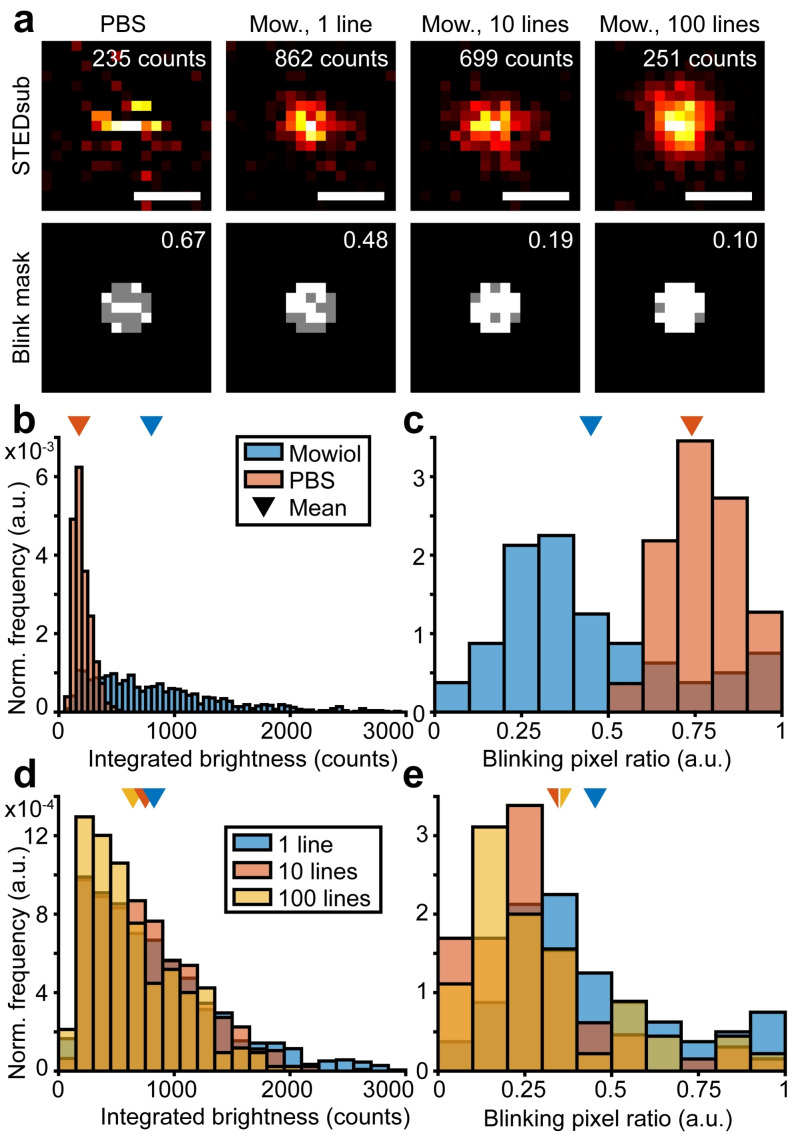
Photophysical characterization of red‐shifted CdTe QDs under STED illumination. (a) Representative STEDsub images of single QDs, with respective blinking mask showing in which pixels the QD is ON (white) or OFF/blinking (grey). Inset numbers show integrated brightness of the QD and blinking pixel ratio. Scale bars, 200 nm. (b, d) Integrated brightness (b) and blinking pixel ratio (d) for QDs in Mowiol (blue) and PBS (orange). (c, e) Integrated brightness (c) and pixel blinking ratio (e) for QDs in Mowiol, for various image acquisition modes; 1 line×200 μs (blue), 10 lines×20 μs (orange), and 100 lines×2 μs (yellow). N=1169 (Mowiol, 1 line×200 μs), N=875 (PBS), N=829 (10 lines×20 μs), and N=283 (100 lines×2 μs). Mean values are marked with arrows.

In images recorded with a single line scan (Figure 1), dark lines are visible on single QDs along the fast scanning axis. These indicate blinking events, where for μs–ms periods a QD can enter a metastable dark state.[^24^, ^25^, ^26^, ^27^] We see that this is stronger in the confocal image (Figure 1c, Confocal) than the STED images (Figure 1c, Exc+STED, STEDonly), indicating that the depletion beam suppresses blinking as previously reported.^[15]^ However, there is still residual blinking, and the effect of this on the final image can be prominent. The blinking as well as the total brightness of the QDs are greatly affected by the surrounding medium and the acquisition settings (Figure 2). Comparing the blinking pixel ratio calculated from the blinking map of the STEDsub image (Figure 2a, second row, details in Supplementary Note S4) of a QD in PBS (Figure 2a, first column), a buffer solution mimicking the intra‐cellular environment, and Mowiol (Figure 2a, second column), a sample mounting medium, we see a clear difference in the amount of blinking. The Mowiol‐surrounded QD shows less blinking than the QD in PBS, while also exhibiting a higher integrated signal. Indeed, analysing more than 800 QDs in each environment, it is clear that this is a general trend (Figure 2b, c), where the mean blinking pixel ratio is 0.44 for the QDs in Mowiol, and 0.74 for the QDs in PBS, i. e., a mean decrease of 40 % in Mowiol (****, details in Supplementary Note S3) (Figure 2c). For the brightness, the mean integrated signal in Mowiol is 826 counts, while it is 205 counts for the PBS environment, i. e., a mean increase of 403 % in Mowiol (****) (Figure 2b). This difference could be attributed to the different pH of PBS and Mowiol, 7.4 and 8.5 respectively. Varying the pH has previously been shown to affect properties of QDs such as the brightness, and especially that lower pH leads to more blinking and lower brightness, speculated to be due to an increase in metastable and dark surface traps.[^28^, ^29^] Lower integrated brightness is moreover a direct effect of increased blinking in point‐scanning imaging.

To further minimize the effect of blinking on an image of a QD in a Mowiol environment, adding multiple repeated line recordings is a possibility (Figure 2a, column 3 and 4, Figure 2d, e). Recording the same total pixel dwell time (200 μs) split up into 1, 10, or 100 lines gives an effect on both integrated brightness and apparent blinking. As expected, when a line recording is significantly faster than the mean blinking events, the apparent blinking on the final added‐up image is averaged out and reduced if more lines with a shorter pixel dwell time are recorded. This effect significantly reduces the mean blinking pixel ratio from 0.44 to 0.34 or 0.35 when 10 or 100 lines are recorded respectively (****) (Figure 2e). Recording 10 or 100 lines does however not make a difference, indicating that at 10 lines, i. e. a pixel dwell time of 20 μs, we are already probing the dark states in a way that the QD more commonly stay on or off for the duration of a whole line across it (∼0.1 ms). From these results we can assume a dark state lifetime on the order of 0.1–1 ms. The brightness is affected in the opposite way, where the mean is significantly decreasing from 826 counts for a single line recording, to 763 counts (*) and 641 counts (****) for the 10‐ and 100‐line recordings respectively (Figure 2d) (Supplementary Table S1). This is hypothesized to be due to the fact that a blinking event will affect more pixels than if we do a slower scan with a single frame, and hence decrease the brightness in multiple pixels at once. This effect increases even more at 100 lines. Simulations of STED images of QDs closely follows the experimental findings, and confirms that when the line recording is significantly faster than the mean time spent in the blinking state the effect of the blinking on the final image is reduced while the integrated brightness is slightly decreased (Supplementary Note S4 and Supplementary Figure S4). With this knowledge, imaging of QDs was performed by adding up 10 repetitively scanned lines to minimize the apparent blinking but still maximize the signal and optimize the recording time.

**Figure 3:**
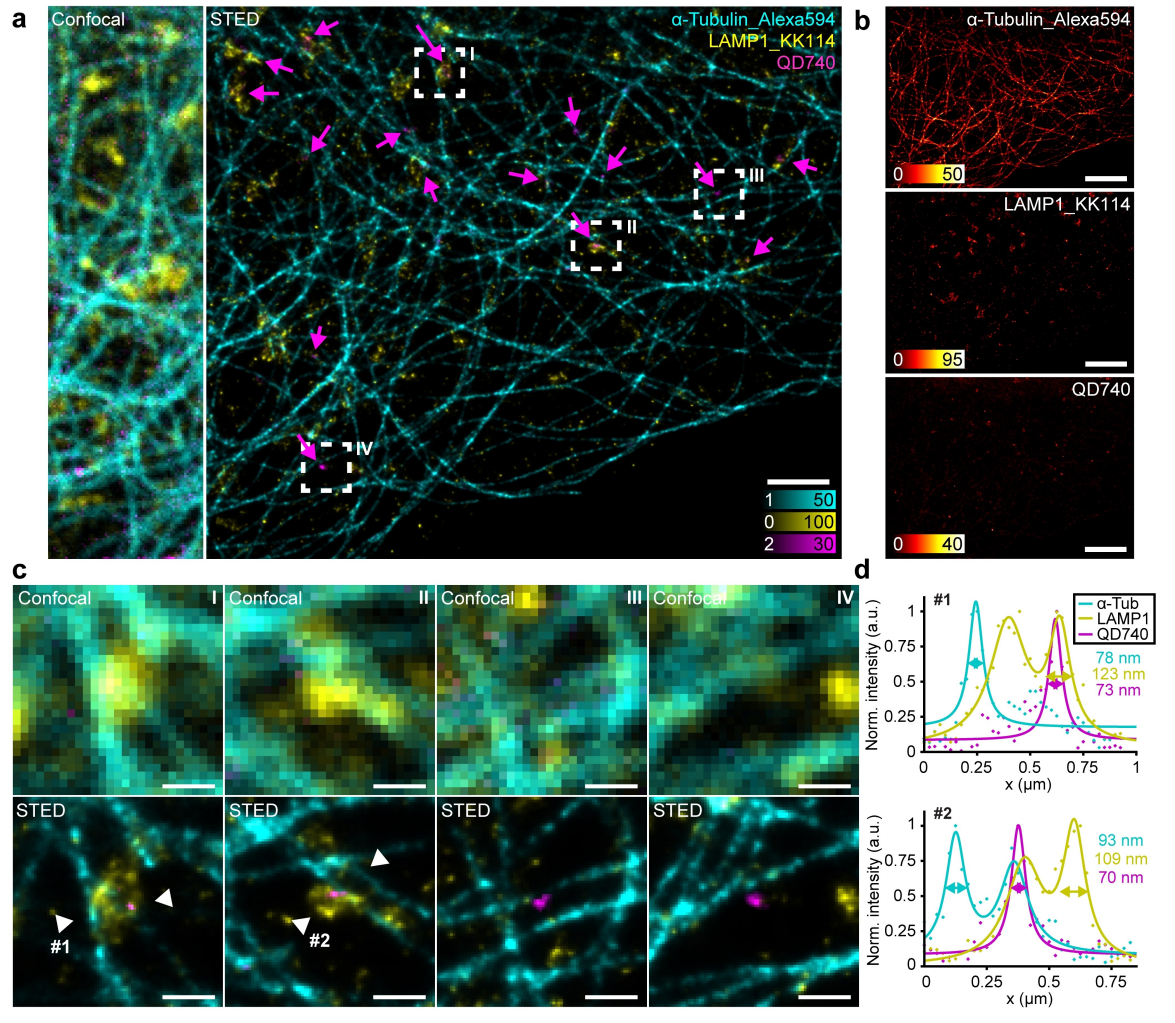
Three‐colour STED imaging of U2OS cells. (a) Three‐colour STED image of LAMP1 labelled with KK114 (yellow), α‐Tubulin labelled with Alexa594 (cyan), and QD740 (magenta) in U2OS cells. QD incubation time: 12 h. Scale bar, 2 μm. (b) Individual channels in (a). Scale bars, 5 μm. (c) Zoom‐ins of the regions of interest I–IV marked in (a), showing confocal and STED images respectively. Scale bars, 500 nm. (d) Line profiles as marked in (c), with raw data of QD740 (magenta dots), α‐Tubulin (cyan dots) and LAMP1 (yellow dots) and fitted single or double Lorentzian profiles (solid lines).

Thanks to the very far red‐shifted emission spectrum and blue‐shifted excitation spectrum the QDs could be imaged as a third channel in three‐colour imaging together with KK114 (also known as Abberior STAR RED) and Alexa594 (Figure 3). U2OS cells were incubated with QDs for 12 h, fixed, and labelled for a lysosomal membrane protein, LAMP1, and tubulin by immunolabelling (Figure 3a). Here, imaging was performed as a two‐step process where KK114 (excitation 640 nm, detection 655–755 nm) and Alexa594 (excitation 561 nm, detection 600–630 nm) were imaged first, and then QD740 (excitation 510 nm, detection 655–755 nm) after a bleaching step. The separation of the spectra however allows the three channels to be recorded in a line‐by‐line‐alternating fashion when using three spectrally separated detectors by dividing the red‐shifted detection around 700 nm. The individual channels in the three‐colour images can be imaged with a certain bleed‐through, as shown (Figure 3b) and further quantified (see Supplementary Note S6 for a further description of the calculations and discussion of the results and see Supplementary Figure S6 for the results). We found that the maximum bleed‐through signal ratio is ∼12 % for any of the fluorophore‐channel combinations, indicating that we can consider a negligible bleed‐through in all channels.

We find QDs inside the cell (Figure 3a, magenta arrows), with one subpopulation located inside LAMP1+ vesicles and another located outside of these vesicles (Figure 3c), indicating that lysosomes are not the only organelles responsible for the uptake of the QDs. Regardless of colocalization with LAMP1+ vesicles, the QDs tend to be in the vicinity of tubulin strands (Figure 3c, ROI III, IV). This further indicates that they are engulfed by vesicles that travel on the microtubule network, suggesting the endocytic pathway with early endosomes, late endosomes and lysosomes as a candidate pathway for the uptake of QDs. The resolution in the multicolour image can be estimated by Lorentzian‐fitted line profiles of the three channels, which shows that we achieve 70 nm‐range FWHMs in all three channels (Figure 3d). It is important to note that the QDs are not always visible in the confocal images (Figure 3c, ROI II–IV). This is due to a combination of the higher blinking rate and faster imaging speed in the confocal images. Thanks to the blinking‐reducing effect of the depletion beam on the QDs and the optimized acquisition settings we can observe, locate, and resolve them clearly in the STED images.

**Figure 4:**
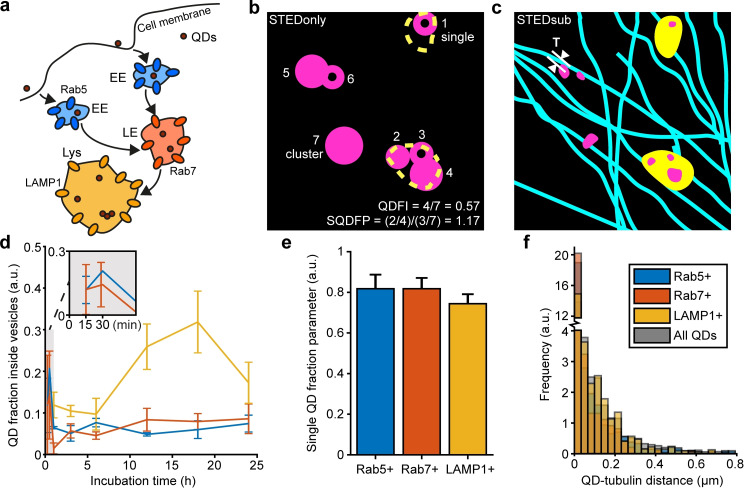
STED investigation of the endocytic pathway for QDs in U2OS cells. (a) Sketch of the endocytic uptake of QDs, showing early endosomes (EE), late endosomes (LE), and lysosomes (Lys). (b) Sketch of a representative STEDsub three‐colour FOV, showing the expected STEDonly image. QDs show up as filled circles (2, 4, 5, 7) or donut‐shaped emissions (1, 3, 6), if it is a cluster or a single QD respectively. Outlines of endocytic vesicles (yellow dashed lines) are marked. Calculated quantities are QD fraction inside vesicles (QDFI) and single fraction parameter (SFP). (c) Sketch of the same FOV as in (b), showing endocytic vesicles (yellow), tubulin (cyan), and QD740 (magenta). Sketch shows distance T from QD to the nearest microtubule. (d, e, f) QDFI, SFP and T plotted for Rab5+ (blue), Rab7+ (orange) and LAMP1+ (yellow) vesicles, and in the case for QDFI for an incubation time from 15 min to 24 h. Number of analysed cells per time point (Rab5+/Rab7+/LAMP1+): 15 min (2/2/–), 30 min (1/2/–), 1 h (6/5/5), 3 h (6/5/5), 6 h (5/5/5), 12 h (8/6/10), 18 h (6/6/6), 24 h (6/10/7). Error bars indicate standard error of the mean.

To further follow the entry of QDs into cells, we studied the endocytic pathway using three‐colour STED (Figure 4). Cells were incubated with QDs for a time ranging between 15 min and 24 h. Following, tubulin as well as a protein specific for one type of endocytic vesicle (Figure 4a, b) were labelled. The three main constituents of the endocytic pathway were investigated: early endosomes (Rab5+), late endosomes (Rab7+) and lysosomes (LAMP1+), and three parameters were quantified: the fraction of QDs inside vesicles (Figure 4b, d), the clustering tendency of QDs (Figure 4b, e), and the distance to the closest microtubule (Figure 4c, f). The fraction of QDs inside vesicles was investigated for different incubation times (Figure 4d). This shows that QDs are endocytosed first by early and late endosomes, as these fractions are increasing and reaches their maxima between 0–30 min. Afterwards, the QD fraction inside lysosomes is steadily increasing at incubation times of 6–18 h, indicating that late endosomes are merging with lysosomal vesicles after ∼6 h. Towards 24 h, the fraction inside lysosomes is decreasing, indicating that QDs are either expelled from the cell or transported to a non‐monitored step in the pathway.

When it comes to the clustering tendency of QDs, the single fraction parameter (SFP) shows the tendency of there being single QDs inside a specific type of vesicle as opposed to those that are outside of the labelled vesicle type (Figure 4e). This is calculated by utilizing the information from the saturated doughnut‐shaped excitation in the STEDonly image, where we expect a central zero intensity for a single QD while a cluster of QDs will have an increased central intensity, despite being unresolvable in the STEDsub image (Supplementary Note S5, Supplementary Figure S5). A higher SFP indicates more single QDs and a lower SFP indicates more clustering in a specific type of vesicle compared to the rest of the cell. The findings here indicate that there is no significant difference in clustering between the different types of vesicles, as the SFP for QDs in early endosomes is 0.82±0.45, late endosomes 0.83±0.33 and lysosomes 0.74±0.31 (all n. s., Supplementary Table S2). It should be noted that previous studies have indicated that gold nanoparticles with a size of 50 nm are clustered more in lysosomes than endosomes.^[30]^ This contrasts with this study and indicates a difference in how the endocytic pathway handles nanoparticles of different sizes and possibly different materials. The smaller‐sized nanoparticles, with a size of 6 nm, instead seems to go through the pathway with a constant degree of clustering along all compartments.

Lastly, the distance between every QD and the closest tubulin strand (Figure 4f) is often less than 200 nm: for 68 % of QDs in early endosomes, 79 % in late endosomes, and 80 % in lysosomes respectively. As previous studies have shown that all endocytic vesicles are transported via the microtubule network,[^31^, ^32^] QDs closer to tubulin can indeed be cargoes. The mean distance between a QD and the closest tubulin is 77 nm (median 0 nm) for QDs in early endosomes, 45 nm (median 0 nm) for QDs in late endosomes, and 87 nm (median 35 nm) for QDs in lysosomes, indicating that lysosomes are the biggest vesicles of the three.

## Conclusions

We presented STED imaging of far red‐shifted core‐only semiconductor QDs in an unexplored spectral window. Upon assessing the performance of the QDs under STED imaging conditions, we determined optimal imaging parameters to minimize blinking effects and maximize apparent brightness.

The far red‐shifted QDs fill a spectral emission gap in the spectrum of commonly used fluorophores for multicolour STED nanoscopy with 775 nm depletion. This enabled to combine QDs and fluorophores to achieve three‐colour STED imaging with a single depletion beam and sub‐70 nm resolution, in a manner that is directly applicable to commercial and custom‐built setups. The proposed multicolour approach features intrinsic channel alignment thanks to the use of a single depletion beam. It is also straightforward as it does not require spectral unmixing or lifetime separation, which can be complicated and potentially error‐prone post‐processing. The full potential of QDs for STED imaging can be reached with functionalization and specific labelling.^[14]^ QDs with directly conjugated antibodies are available commercially^[15]^ from multiple producers, but further developments and availability of similar products will broaden their use for cellular imaging, especially red‐shifted QDs for their use in 775 nm depletion STED imaging, allowing studies to fully benefit from their properties.

In the presented imaging scheme, we also used the raw STEDonly imaging data to extend the information content of the resolved object. By examining the spatial distribution of the fluorescence signal generated by the donut illumination we determined if the observed QDs are single or clustered QDs. Together, this allowed us to look closer at the endocytic pathway of U2OS cells, quantifying the amount of QDs inside the endocytic vesicles and investigating the clustering down the endocytic pathway. Finally, the emission at 740 nm opens new possibilities for STED imaging in deeper tissue with reduced scattering and illumination energy.

## Experimental Section

### STED Microscope

All images were acquired on a modified custom‐built STED setup previously published,^[21]^ schematically drawn in Supplementary Figure S1, and more thoroughly described in Supplementary Note 1. An exhaustive list of all optical elements and devices can be found in Supplementary Note 2.

In short, excitation of the QDs and dyes was performed with three pulsed diode lasers at 510 nm, 561 nm, and 640 nm, each with pulse widths of <130 ps and repetition rates of 40 MHz. The depletion was performed with a laser at 775 nm with a pulse width of ∼530 ps, phase‐shaped into a donut using a spatial light modulator. The beams are scanned across the sample using galvanometric mirrors, and the objective lens used is a 100×/1.4 NA oil immersion objective (HC PL APO 100×/1.40 NA Oil STED White, Leica Microsystems). Dichroic mirrors are used to combine laser lines and separate the fluorescence light from incoming laser beams, and finally bandpass filters are used to collect the signal on two APD detectors, at 655–755 nm and 600–630 nm. The microscope was controlled using a NI‐DAQ acquisition board and two separate software: Imspector^[33]^ (Max‐Plank Innovation) and custom‐written open‐source software Tempesta (https://github.com/jonatanalvelid/Tempesta‐RedSTED; now ImSwitch,^[34]^ https://github.com/kasasxav/ImSwitch).

### STED Imaging and Acquisition Settings

Images for the photophysical investigation of the QDs were recorded using various acquisition settings, setting the single line pixel dwell time to 2 μs, 20 μs, or 200 μs, and repeating each line 100×, 10×, or 1×, always totalling a pixel dwell time of 200 μs.

Three‐colour STED images were recorded as a combination of: (1) a line‐by‐line single line scan with a dwell time of 30 μs for the two channels of fluorescent dyes, alternating depletion laser power with the AOM and the detector read‐out, and (2) a repeated line scan with 10 repetitions of a pixel dwell time of 5 μs of the QDs. The last image was recorded immediately after a bleaching step where all the lasers were turned on in order to bleach the remaining fluorescent dyes. This step is possible thanks to the highly photostable QDs, but is not necessary if using three spectrally separated detectors simultaneously.

STED images were recorded using a pixel size of 25 or 30 nm, with a pixel dwell time of 2–200 μs. Image sizes were 10×10 μm^2^ for the QD characterization, and 30×30 μm^2^ for the three‐colour STED images. For the QDs, the 510 nm excitation power was 5–10 μW, the 775 nm depletion power was 122–147 mW, and the signal was detected in the 655–755 nm channel. For Alexa594, the 561 nm excitation power was 4.8–8.1 μW, the 775 nm depletion power was 109–110 mW, and the signal was detected in the 600–630 nm channel. For KK114, the 640 nm excitation power was 1.4–4.3 μW, the 775 nm depletion power was 65–94 mW, and the signal was detected in the 655–755 nm channel. All laser powers were measured at the conjugate back focal plane of the objective lens, between the scan and the tube lens.

All images presented are raw, unprocessed data, unless otherwise stated. To aid visualization, the STEDsub image in Figure 1a as well as three‐colour STED images in Figure 3a and Supplementary Figure S2 have been deconvolved with a Richardson‐Lucy deconvolution algorithm (Imspector), using a Lorentzian PSF with a FWHM of 30–50 nm. All line profiles and data collection has been done on the raw unprocessed images, regardless of the presented version of the image.

### Quantum Dots

The QDs, QD740, used are hydrophilic CdTe QDs (PL‐QDN‐740, PlasmaChem GmbH). They are core‐only QDs terminated with carboxyl groups. The diameter of the CdTe core is 6.0 nm, and the molecular weight is 380 kDa. The emission maximum is at 744±1 nm and the spectral width is 73±1 nm, both extracted from a Gaussian fit (R^2^=0.997) of the emission spectrum. The dry powder of QDs is dissolved in Milli‐Q water and kept in a stock solution of 125 μM.

### Cell Culture and Sample Preparation

U2OS cells (ATCC HTB‐96) were cultured in a humidified incubator at 37 °C and 5 % CO_2_ in Dulbecco's modified Eagle medium (DMEM) (41966029, Thermo Fisher Scientific) supplemented with 10 % (vol/vol) fetal bovine serum (10270106, Thermo Fisher Scientific) and 1 % Penicillin‐Streptomycin (P4333, Sigma‐Aldrich). Cells were seeded on coverslips in a six‐well plate, and later fixed and labelled. Fixation was done using 4 % PFA in PBS for 10 min, permeabilized by incubation with 0.5 % TRITON X100 in PBS for 5 min and after blocked with 5 % BSA in PBS for 30 minutes. Cells were then incubated with a mixture of the primary antibodies in BSA in PBS for 1 hour and after incubated with a mixture of secondary antibodies in BSA and PBS for 1 hour. Finally, the cells on the cover slips were mounted in Mowiol mounting medium. Washing with PBS was performed between each step, and all the process was performed at room temperature.

Before the fixation and labelling process, the cells were incubated with QDs for 15 min, 30 min, 1 h, 3 h, 6 h, 12 h, 18 h or 24 h. A small volume of a solution of the QDs in Milli‐Q water was added to the normal cell culture medium to reach a final concentration of 3.75 nM (1 : 3.3×10^4^ of stock solution), and cells were left to incubate at 37 °C for an amount of time as indicated above.

Samples with only QDs for the QD characterization was prepared by putting a 0.50 nM solution of QDs in Milli‐Q water on a cover slip, leaving it until the Milli‐Q water evaporated, and then mounting the cover slip with either Mowiol or PBS.

## Author Contributions

I. T. designed and supervised the research and conceived the project idea. J. A. planned and built the setup; planned, prepared, and performed experiments; developed analysis pipelines; and analysed data. A. B. developed analysis pipelines and analysed data. J. A. and I. T. wrote the manuscript with input from all the authors.

## Conflict of interest

The authors declare no conflict of interest.

1

## Supporting information

As a service to our authors and readers, this journal provides supporting information supplied by the authors. Such materials are peer reviewed and may be re‐organized for online delivery, but are not copy‐edited or typeset. Technical support issues arising from supporting information (other than missing files) should be addressed to the authors.

Supporting Information

## Data Availability

Code of analysis and simulation scripts together with example data supporting the findings of this study are publicly available on GitHub and Zenodo; repository: https://github.com/jonatanalvelid/qdsted‐analysis‐simulations, Zenodo: https://doi.org/10.5281/zenodo.7081736. Additional data supporting the findings of this study are available from the authors upon request.
