# Supplementary material for: Far Red‐Shifted CdTe Quantum Dots for Multicolour Stimulated Emission Depletion Nanoscopy
Source: Chemphyschem. 2022 Nov 9;24(3):e202200698. doi: 10.1002/cphc.202200698 (PMC10098508; doi:10.1002/cphc.202200698)
Supplement: Supplementary file 1 — Supporting Information [file CPHC-24-0-s001.pdf]

ChemPhysChem

Supporting Information

Far Red-Shifted CdTe Quantum Dots for Multicolour Stimulated Emission Depletion Nanoscopy

Jonatan Alvelid, Andrea Bucci, and Ilaria Testa*

Supplementary Information

List of Supplementary Notes

S1. STED microscope
S2. List of setup components
S3. Photophysical characterization of QDs
S4. Simulation of QD photophysical characterization
S5. Cluster detection and endocytosis pathway quantification
S6. Bleed-through ratio calculations for fluorophores

List of Supplementary Figures

S1. STED images of QDs in various environments and with different acquisition settings.
S2. Three-colour STED imaging of U2OS cells.
S3. Custom-built STED setup used for imaging.
S4. Simulation of STED imaging affected by QD photophysics
S5. Simulated and experimental STEDsub and STEDonly imaging of single QDs and QD clusters
S6. Bleed-through signal ratio for fluorophores in channels

List of Supplementary Tables

S1. Results of Kolmogorov-Smirnov statistical tests between photophysics characterization datasets.
S2. Results of Kolmogorov-Smirnov statistical tests between QD endocytosis datasets.

1. Supplementary Notes

Supplementary Note S1. STED microscope

All images were acquired on a modified custom-built STED setup previously published[1], schematically drawn in Supplementary Fig. S3. Excitations of the QDs and dyes were done with three pulsed diode lasers, one at 510 nm (LDH-D-C-510), the second at 561 nm (PDL561, Abberior Instruments), and the third at 640 nm (LDH-D-C-640, PicoQuant). The 510 nm laser has a pulse width of < 130 ps, while the 561 nm and 640 nm lasers have pulse widths of < 60 ps. The 561 nm and 640 nm excitation laser beams are coupled into a fibre (P5-488PM-FC-2, Thorlabs) to co-align the beams and shape the wave fronts. The 775 nm depletion laser beam (KATANA 08 HP, OneFive), with a pulse width of 530 ps, is led through an AOM (MT110-B50A1.5-IR-Hk + MDS1C-B65-34-85.135-RS, AA Opto Electronic) for line-by-line power modulation between the channels and then coupled into a polarization-maintaining single-mode fibre (PMJ-3AHPM3S-633-4/125-3-3-1, OZ Optics). One polarization direction of the beam is extracted with an interferometer-like arm, and subsequently shaped using a vortex phase mask on a spatial light modulator (LCOS-SLM X10468-02, Hamamatsu Photonics). The three excitation and one depletion beams are merged into a single beam path through three dichroic mirrors. They pass through a λ/4 wave plate and a λ/2 wave plate to create the circular polarization necessary for optimal depletion doughnut focus formation and are subsequently scanned using fast galvanometer mirrors (galvanometer mirrors 6215H + servo driver 71215HHJ 671, Cambridge Technology). The objective lens used is a 100×/1.4 NA oil immersion objective (HC PL APO 100×/1.40 NA Oil STED White, 15506378, Leica). The fluorescence is collected back through the same beam path, and is de-coupled from the incoming laser beams through a multi-bandpass dichroic mirror (ZT405/488/561/640/775rpc, Chroma Technology) and a long-pass dichroic mirror (ZT514RDC, Chroma Technology) after being de-scanned by the galvanometer mirrors. The combined fluorescence signal is then passed through a pinhole with a diameter of 75 µm (1.28 Airy disk units) and separated with another dichroic mirror (Di02-R635, Semrock) into two channels. Channel 1 (red) has a notch filter (NF03-785E-25, Semrock) and a bandpass filter (ET705/100m, Chroma Technology), and the fluorescence is focused onto a free space APD (SPCM-AQRH-13-TR, Excelitas Technologies). Channel 2 (green) has a notch filter (ZET785NF, Chroma Technology) and a bandpass filter (ET615/30m, Chroma Technology), and the fluorescence is focused onto a 62.5 µm core diameter multi-mode fibre (M31L01, Thorlabs) coupled to an APD (SPCM-AQRH-14-FC, PerkinElmer). The fluorescence signal is collected by a NI-DAQ acquisition board (PCIe-6353, National Instruments), also used to control the lasers and the scanning. Using an infrared laser in total internal reflection, a feedback-loop-based focus lock is additionally used in the microscope, keeping the sample in the desired focal plane during acquisitions.

The microscope was controlled through two separate software: image acquisition and some hardware control were done through the Imspector software (Max-Planck Innovation, Göttingen, Germany) while the rest of the hardware control (SLM, focus lock and 775 nm laser output power) was done through Python-based custom-written microscope control software Tempesta (https://github.com/TestaLab/Tempesta, https://github.com/jonatanalvelid/Tempesta-RedSTED; now ImSwitch[2],

https://github.com/kasasxav/ImSwitch). The 775 nm laser acts as the master trigger in the system, pulsing at 40 MHz, and triggers the three excitation lasers through three separate picosecond delayers, two commercial (PSD-065-A-MOD, Micro Photon Devices) and one home-built. This allows for picosecond-level control of the delay between the excitation pulses and depletion pulses, which is crucial to optimize the STED efficiency and hence resolution and image quality for specific fluorophores.

Supplementary Note S2. List of setup components

Lenses: L1: 200 mm, L2: 100 mm, L3: 200 mm, L4: 400 mm, L10: 150 mm (all AC254-XXX-B-ML, Thorlabs), L5: 100 mm, L6: 200 mm, L7: 200 mm, L8: 100 mm, L9: 200 mm, L11: 30 mm (all AC254-XXX-A-ML, Thorlabs), SL: 50 mm (Leica Microsystems), TL: 200 mm (Leica), OBJ: HC PL APO 100x/1.40 Oil STED White (15506378, Leica). **Filters**: BPF1: ET705/100m (Chroma Technology), BPF2: ET615/30m (Chroma), NF1: NF03-785E-25 (Semrock), NF2 and NF3: ZET785NF (Chroma), CUF: CT780/20bp (Chroma). **Dichroic mirrors**: DM1: T610spxxr (Chroma), DM2: T700dcspxruv_UF3 (Chroma), DM3: ZT405/488/561/640/775rpc (Chroma), DM4: T860SPXRXT (Chroma), DM5: ZT514RDC (Chroma), DM6: Di02-R635 (Semrock). **Lasers**: 510: LDH-D-C-510 (510 nm, PicoQuant), 561: PDL561 (561 nm, Abberior Instruments), 640: LDH-D-C-640 (640 nm, PicoQuant), 775: KATANA 08 HP (775 nm, OneFive), 980: CP980S (Thorlabs). **Detectors/cameras**: APD1: SPCM-AQRH-13-TR (Excelitas Technologies), APD2: SPCM-AQRH-14-FC (PerkinElmer), CMOS: DMK 33UP1300 (The Imaging Source Europe). **Scanners**: GX/GY: 6215H Galvanometric mirrors + 71215HHJ 671 Servo Driver (Cambridge Technology), PZ: Z-piezo stage LT-Z-100 (Piezoconcept). **Fibre optics**: F1: PMJ-3AHPM3S-633-4/125-3-3-1 (OZ Optics), F2: P5-488PM-FC-2 (Thorlabs), F3: M31L01 (Thorlabs), FC1: 60SMS-1-0-A2-02, FC2: 60SMS-1-0-A18-02, FC3: 60SMS-1-4-M5-33 (Schäfter + Kirchhoff). **Misc.**: PH: P75H (Thorlabs), AP: D15S (Thorlabs), PBS: PTW 1.15 (B.Halle Nachfl.), λ/4-1: 460-680 achr. (RAC 3.4.15, B.Halle Nachfl.), λ/4-2: 500-900 achr. (RAC 4.4.15, B.Halle Nachfl.), λ/2: 500-900 achr. (RAC 4.2.15, B.Halle Nachfl.), SM: 50 mm concave mirror (CM508-050-P01, Thorlabs), SLM: LCOS-SLM X10468-02 (Hamamatsu Photonics), AOM: MT110-B50A1.5-IR-Hk + MDS1C-B65-34-85.135-RS (AA Opto Electronic), M: BB1-E02 (Thorlabs), all other mirrors: PF10-03-P01 (Thorlabs).

Supplementary Note S3. Photophysical characterization of QDs

Photophysical characterization of quantum dots and comparison between different environments and acquisition settings were always done at identical excitation power, as measured in the conjugate back focal plane, to achieve fully comparable measurements.

For the photophysical characterization and specifically the blinking pixel ratio, a blinking mask is automatically calculated for every QD from the raw image. It is determined by comparing the STEDsub image with an average STEDsub image of all the detected single QDs in the whole image. The pixels where the QD is unusually dark compared to the averaged image (less than the average with the Poisson noise subtracted) are marked as blinking pixels. The rest of the pixels are considered as bright pixels, inside a radius equal to the distance to the furthest bright pixel that is above the background Poisson noise level (most of the time 3 pixels, i.e. a diameter of 7 pixels or 210 nm when the pixel size is 30 nm).

The statistical significance of the difference in the blinking and brightness between the different environments and acquisition settings were tested with two-sample two-sided Kolmogorov-Smirnov tests. These tests test the null hypothesis that two independent random samples are drawn from the same underlying distribution. The test takes on a standard 5% significance level for rejecting the null hypothesis. The results from the statistical tests are shown in Supplementary Table S2. For the blinking parameter the tests were two-sided, while for the brightness parameter the tests were one-sided (smaller tail), as the brightness distributions all have similar mode values but different higher value tail shape. When statistical test results are mentioned, the following symbols are used: n.s. ($p > 0.05$); * ($p < 0.05$); ** ($p < 0.01$); *** ($p < 0.001$); **** ($p < 0.0001$).

Supplementary Note S4. Simulation of QD photophysical characterization

In order to test the results of the QD photophysical characterization for various acquisition settings, QD photophysics and their influence on the STED image under different acquisition settings was simulated. Examples of the simulated images for the different settings, together with distributions of summed intensities and blinking pixel ratios, can be found in Supplementary Fig. S4.

The quantum dot images were simulated in 3 steps, starting with a simulated ideal QD image that then was modified by a blinking mask and lastly background was added. The ideal QD image took the brightness, imaged quantum dot size, pixel size, and pixel dwell time into account. A Boolean blinking mask with the same size as the QD image was created by simulating blinking along each line of imaging, taking the pixel dwell time into account. The blinking was simulated using parameters for mean time in a bright state, mean time in a blinking state, and standard deviation of the mean times. For each line, a randomized starting state (bright or blinking) was assumed, and a line length of 8× the length of the image was simulated from which the middle part was finally taken. The blinking mask was multiplied with the ideal QD image. Finally, a Poissonian background image with a parameter of the mean background count rate was calculated, simulating detector dark counts and any other uniform background, and added to the blinking QD image.

The three acquisition settings tested were pixel dwell times of 2, 20, and 200 µs, with 100, 10, and 1 line repetitions respectively. Simulating 1000 quantum dot images in each case and then calculating the blinking pixel ratio and brightness in the same way as in the real QD images gave the following results. For the brightness, the mean value was 814 counts, 568 counts, and 466 counts for 1, 10, and 100 line repetitions respectively (all ****), with a narrower distribution with more line repetitions. This agrees with the experimental results of significantly reduced counts with an increased number of line repetitions. For the blinking pixel ratio, the mean value was 0.46, 0.32, and 0.19 for 1, 10, and 100 line repetitions respectively (all ****), also with a narrower distribution with more line repetitions. Here, the significant reduction from 1 to 10 lines in the real experiments is reproduced, while additionally 100 line repetitions reaches an even lower blinking pixel ratio. This small disparity can likely be explained by a simplified simulation, assuming both a blinking model without the full complexity of real quantum dots and a simple background model where only a uniform

background contribution is assumed. Nonetheless, the general decrease of both intensity and blinking pixel ratio with an increased number of line repetitions is reproduced and confirmed.

The parameter values used for the above simulations were manually tuned for simulated images to be in accordance with experimental images. Importantly, the mean bright and blinking state times used were 1000 μs and 700 μs respectively, with a standard deviation of the times of 2000 μs.

Supplementary Note S5. Cluster detection and endocytosis pathway quantification

To investigate the tendency of QDs to be alone inside vesicles the single fraction parameter (SFP) was calculated. It can be calculated using both the STEDsub image and the STEDonly image. Since each QD gives rise to a doughnut-shaped emission in the STEDonly image, looking at the central intensity of each doughnut allows us to categorize seemingly single QDs in the STEDsub image into single QDs or clusters. If we have a single QD, this central intensity should be zero, while a cluster of QDs should mean that we have some intensity also in the centre of each doughnut from the surrounding QDs. An example of this can be seen in Fig. S5, both in simulated data (Fig. S5a) and experimental data (Fig. S5b). STEDsub has been simulated as the convolution between the sample and a Gaussian beam with a FWHM of 40 nm, corresponding to the effective PSF in STED imaging. STEDonly has been simulated as a saturated excitation with the doughnut-shaped 775 nm depletion beam, with parameters to reach similar saturation levels as seen in experimental data. The QDs are simulated as objects with a side length of 6 nm, and in the cluster the 9 QDs are spaced with a distance equal to 3× the side length. From the simulations and experiments it is clear in the STEDonly images that the central zero intensity in the saturated doughnut-shaped excitation increases if we have a cluster of QDs, as compared to a single QD. Instead, in the STEDsub images, we cannot see a difference between single QDs and clusters of QDs. The differences between the simulated and experimental STEDonly images can be explained by the lack of blinking and other noise-sources in the simulation, however the general shape of the imaged QDs should be unaffected by this.

The SFP is the ratio between the tendency of a QD to be alone inside a vesicle and the tendency of a QD to be alone in the whole image or cell (equation 1):

$$SFP = \frac{N_{single\ QDs\ in\ vesicles}}{N_{QDs\ in\ vesicles}} / \frac{N_{single\ QDs\ in\ cell}}{N_{QDs\ in\ cell}}$$

Comparing the vesicle-specific ratio in the numerator with the whole-cell ratio in the denominator the SFP effectively normalizes it against the number of QDs inside the cell, rendering it a better parameter to compare cells that have been incubated for 15 min or 24 h and therefore likely contain different amount of QDs.

Supplementary Note S6. Bleed-through ratio calculations for fluorophores

The bleed-through signal ratios for the three fluorophores in the three recording channels used in the three-colour imaging have been quantified by looking at how much signal has bled through from each fluorophore to each recorded channel. As previously described[3], the bleed-through ratio, r_{ij}, for fluorophore i in channel j in multi-colour images can be calculated as the

ratio between the total signal S in an area A containing only fluorophore i in the channel dedicated to record fluorophore j and the channel dedicated to record fluorophore i:

$$r_{ij} = S_{ij}/S_{ii}.$$

In our images, the analysis firstly finds the area A_i to consider for each fluorophore i, i.e. the area in the image which is occupied only by a single fluorophore species, by looking at the binarization of the channels with two different thresholds, generating the binary images $b_{i,hi}$ and $b_{i,lo}$ for each channel dedicated to record fluorophore i. The two thresholds are found as two different weightings of a common automatically calculated threshold such as Otsu's threshold or the triangle threshold, where the choice is depending on the structure. The area to consider for fluorophore i is found as:

$$A_i = b_{i,hi} \wedge \neg(b_{m,lo} \vee b_{n,lo}),$$

where m and n are the two other fluorophores, $\wedge$ is the logical AND, $\neg$ is the logical NOT, and $\vee$ is the logical OR operator. The signal S_{ij} is found as the sum in the area A_i in channel j divided by the pixel dwell time of channel j, where the last division is performed in order to normalize for that acquisition parameter. The ratios can then be calculated for the three fluorophores as described above. The code to perform the bleed-through calculations in the three-colour images is found as part of the openly available code repository on GitHub and Zenodo, as mentioned in the *Data and code availability statement*.

The ratio calculation is significantly affected by noise and background values, as the extraction of S_{ij} becomes increasingly dependent on the noise and background in channel j the lower the actual bleed-through is. Furthermore, overlapping structures of different labelling or brightness differences between the dyes will be influencing the extracted signals and bleed-through ratios. Hence, calculated bleed-through ratios should be seen as conservative upper limits.

The results, for N = 7 images, are shown in Supplementary Fig. S6. They show that the bleed-through is found to be negligible in all cases, at a maximum of ~ 12% for the bleed-through of Alexa594 into the KK114 channel, which in the final images is barely noticeable thanks to the relatively strong KK114 signal. Important to note is the bleed-through of QDs into the two other channels; (1) 561 excitation and 600–630 nm detection, and (2) 640 nm excitation and 655–755 nm detection. Here we note that they are found to be (1) 10% and (2) 6% on average, and are not higher since (1) QD emission in the 600–630 channel is low, and (2) QD excitation by 640 nm is low. The strong dependency on noise, background, and overlapping structures can be seen from the large standard deviations, as the values should be constant in ideal imaging conditions.

Supplementary References

1. Alvelid, J. & Testa, I. Stable stimulated emission depletion imaging of extended sample regions. *J. Phys. Appl. Phys.* **53**, 024001 (2019).
2. Casas Moreno, X., Al-Kadhimi, S., Alvelid, J., Bodén, A. & Testa, I. ImSwitch: Generalizing microscope control in Python. *J. Open Source Softw.* **6**, 3394 (2021).
3. Winter, F. R. *et al.* Multicolour nanoscopy of fixed and living cells with a single STED beam and hyperspectral detection. *Sci. Rep.* **7**, (2017).

Supplementary Figures

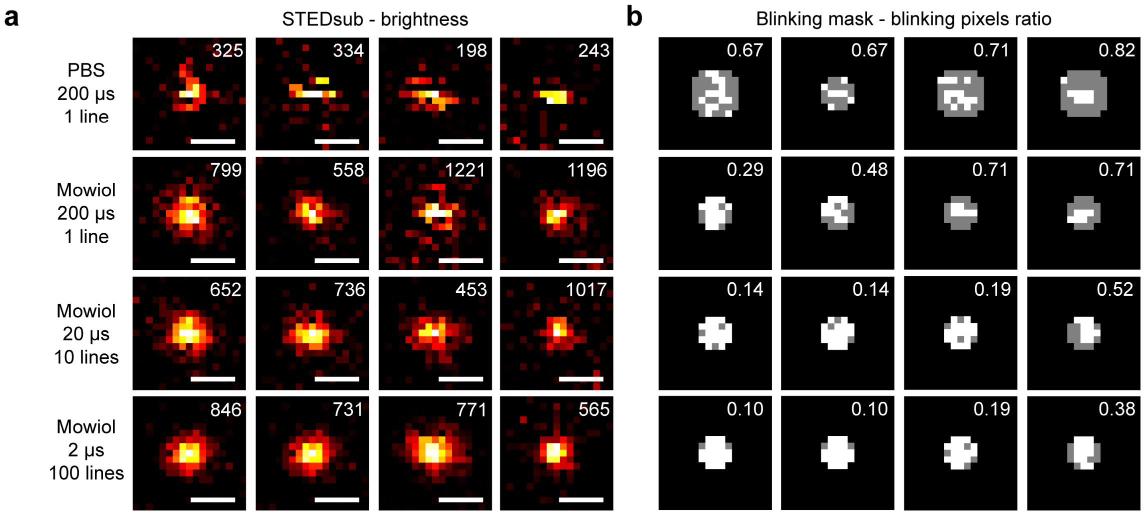

Supplementary Figure S1. STED images of QDs in various environments and with different acquisition settings. (a,b) Four STEDsub images (a) and four blinking masks (b) of QDs in PBS (first line), Mowiol with a single line and a pixel dwell time of 200 µs acquisition (second line), Mowiol with 10 added lines and a pixel dwell time of 20 µs (third line), and Mowiol with 100 added lines and a pixel dwell time of 2 µs (fourth line). Numbers in images indicate integrated counts or brightness (a) and blinking pixel ratio (b) respectively. Scale bars are 200 nm.

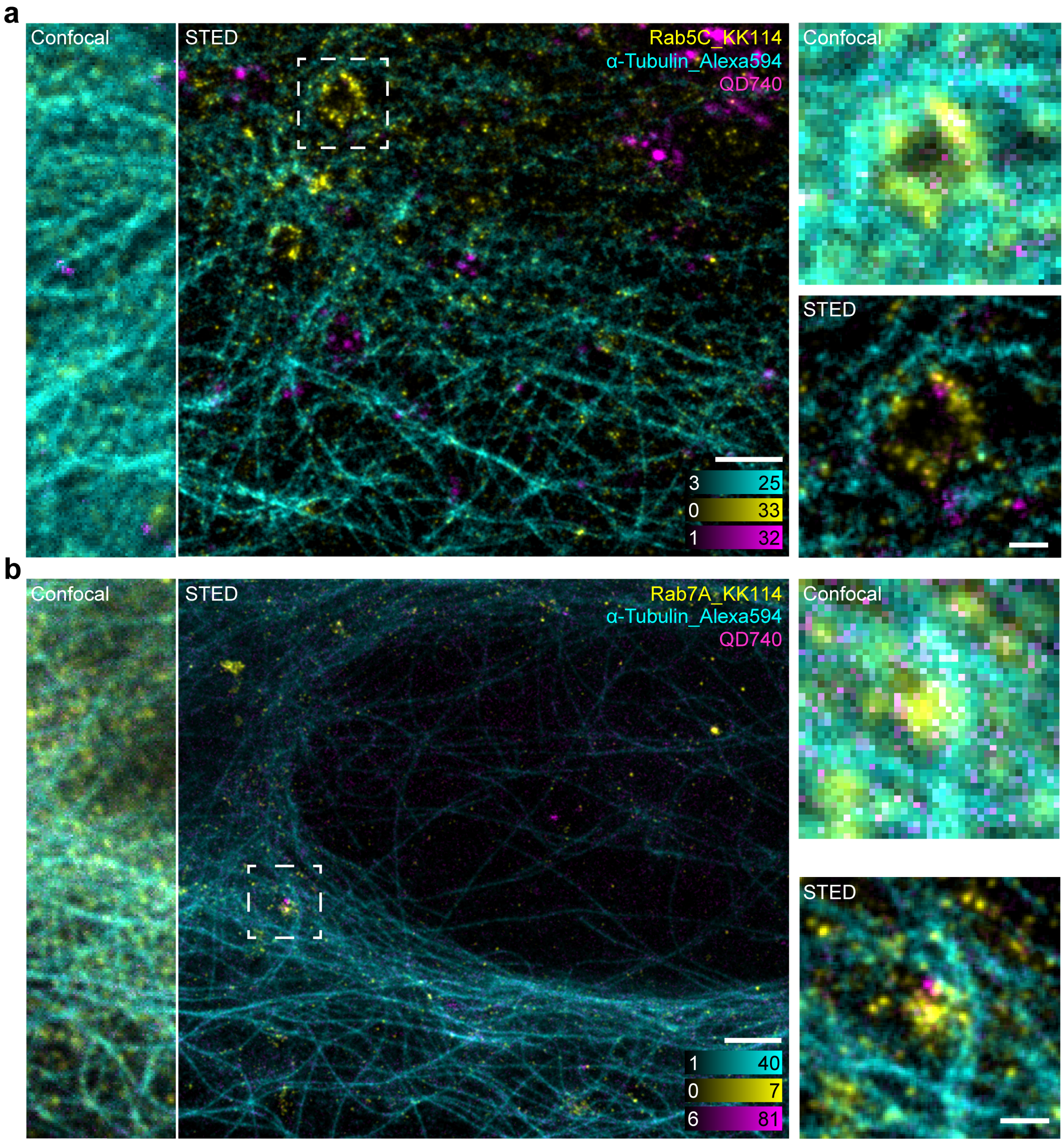

Supplementary Figure S2. Three-color STED imaging of U2OS cells. (a) Three-color STED image of Rab5C stained with KK114 (yellow), α-Tubulin stained with Alexa594 (cyan) and QD740 (magenta) in U2OS cells. Cells were incubated for 18 h after adding QDs to the media before fixation. Scale bars 2 µm (large), 500 nm (zoom-in). **(b)** Three-color STED image of Rab7A stained with KK114 (yellow), α-Tubulin stained with Alexa594 (cyan) and QD740 (magenta) in U2OS cells. Cells were incubated for 24 h after adding QDs to the media before fixation. Scale bars 2 µm (large), 500 nm (zoom-in).

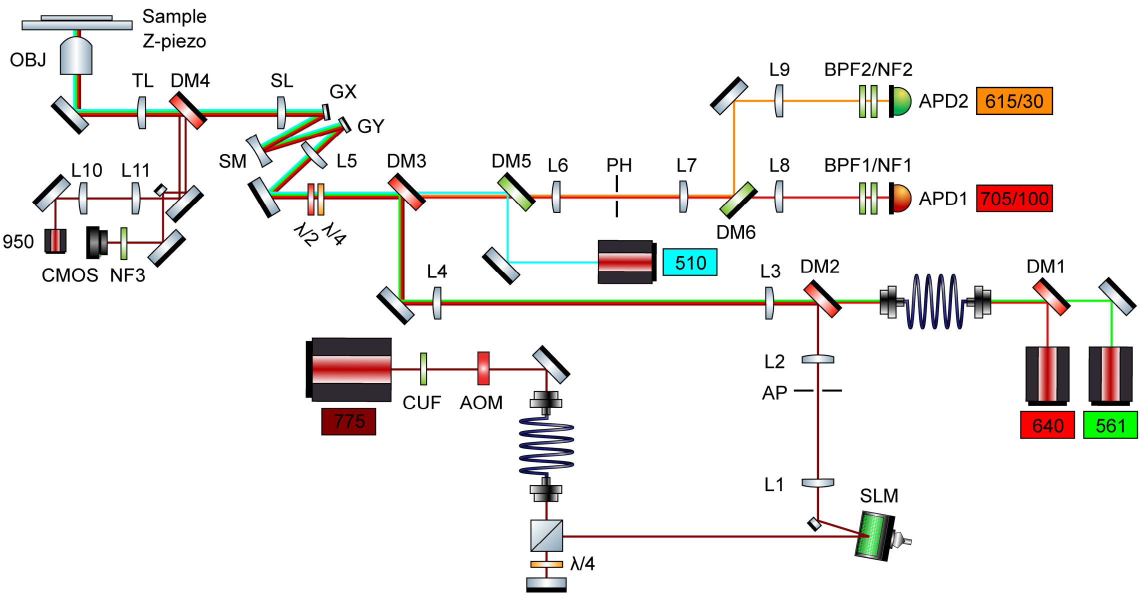

Supplementary Figure S3. Custom-built STED setup used for imaging. Elements from TL to sample are mounted in a Leica DMi8 microscope stand.

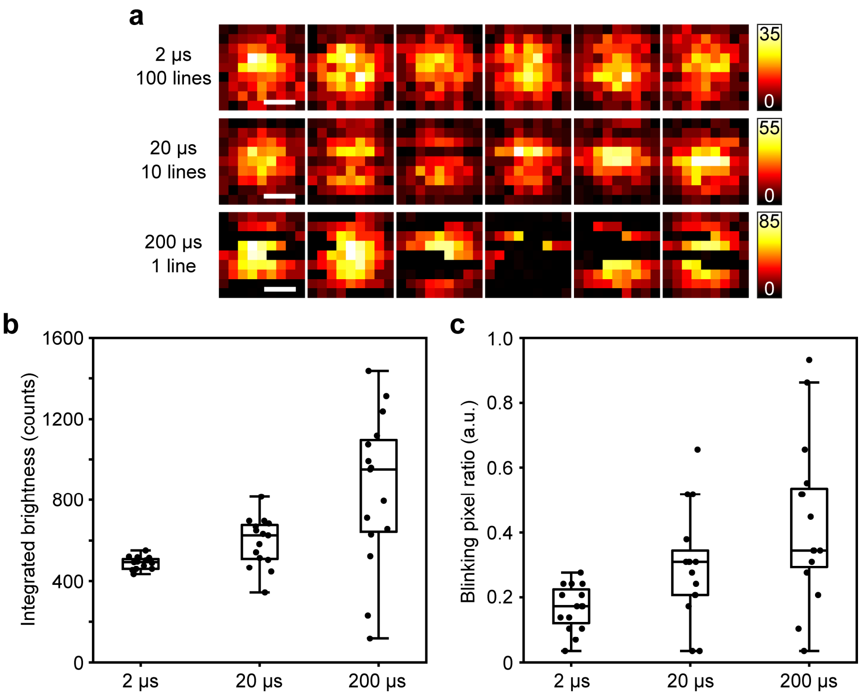

Supplementary Figure S4. Simulation of STED imaging affected by QD photophysics. (a) Example images from simulations with 100 × 2 µs (top row), 10 × 20 µs (middle row), and 1 × 200 µs (bottom row) line repetition/pixel dwell time acquisition settings. **(b)** Box plots of QD integrated intensities for the three acquisition settings. **(c)** Box plots of QD blinking pixel ratios for the three acquisition settings. Scale bars are 100 nm.

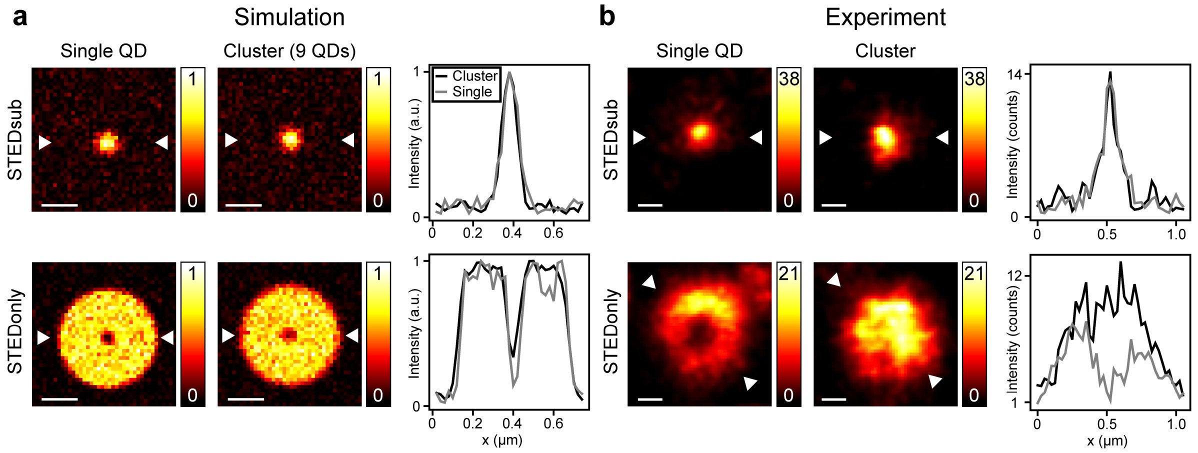

Supplementary Figure S5. Simulated and experimental STEDsub and STEDonly imaging of single QDs and QD clusters. (a) Simulated STEDsub (top row) and STEDonly images (bottom row) of a single QD (left) and a QD cluster (middle), with line profiles across each image (right) showing clusters (black) and single QDs (gray). **(b)** Experimental STEDsub (top row) and STEDonly images (bottom row) of a single QD (left) and a QD cluster (middle), with line profiles across each image (right) showing clusters (black) and single QDs (gray). Scale bars are 200 nm.

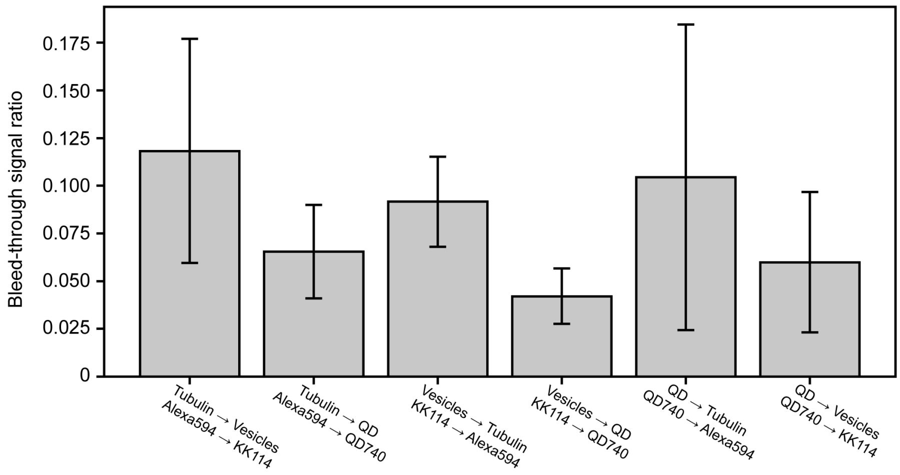

Supplementary Figure S6. Bleed-through signal ratio for fluorophores in channels. Channel bleed-through ratio r_{ij} for fluorophore i in channel j is calculated in three-color STED images as the ratio r between the total signal S in an area containing only fluorophore i in the channel dedicated to record fluorophore j and the channel dedicated to record fluorophore i; $r_{ij} = S_{ij}/S_{ii}$. N = 7 images, error bars show 1 standard deviation.

Supplementary Tables

Supplementary Table S1. Results of two-sample two-sided Kolmogorov-Smirnov statistical tests between photophysics characterization datasets.

Parameter	Dataset 1	Dataset 2	Result	p-value
Blinking	PBS	Mowiol	****	$< 1\times10^{-4}$
Blinking	1 line	10 lines	****	$< 1\times10^{-4}$
Blinking	1 line	100 lines	****	$< 1\times10^{-4}$
Blinking	10 lines	100 lines	n.s.	0.84
Brightness	PBS	Mowiol	****	$< 1\times10^{-4}$
Brightness	1 line	10 lines	*	0.039
Brightness	1 line	100 lines	****	$< 1\times10^{-4}$
Brightness	10 lines	100 lines	****	$< 1\times10^{-4}$

Supplementary Table S2. Results of two-sample two-sided Kolmogorov-Smirnov statistical tests between QD endocytosis datasets.

Parameter	Dataset 1	Dataset 2	Result	p-value
SFP	EE	LE	n.s.	0.27
SFP	EE	Lys	n.s.	0.19
SFP	LE	Lys	n.s.	0.23
